# The trust and insurance models of healthcare purchasing in the Ayushman Bharat Pradhan Mantri Jan Arogya Yojana in India: early findings from case studies of two states

**DOI:** 10.1186/s12913-022-08407-2

**Published:** 2022-08-18

**Authors:** Kheya Melo Furtado, Arif Raza, Devasheesh Mathur, Nafisa Vaz, Ruchira Agrawal, Zubin Cyrus Shroff

**Affiliations:** 1 Goa Institute of Management, Poriem, Sattari, Goa India 403505; 2 National Health Authority, Ministry of Health and Family Welfare, Government of India, New Delhi, India; 3 Alliance for Health Policy and Systems Research, World Health Organization, Geneva, Switzerland

**Keywords:** Strategic purchasing, Provider contracting, Claim management, Purchasing model, Health insurance, Health financing, Universal health coverage, PMJAY, India

## Abstract

**Background:**

The Pradhan Mantri Jan Arogya Yojana (PMJAY), a publicly funded health insurance scheme for the poor in India, was launched in 2018. Early experiences of states with various purchasing arrangements can provide valuable insights for its future performance. We sought to understand the institutional agencies and performance of the trust and insurance models of purchasing with respect to; a) Provider contracting b) Claim management c) Implementation costs.

**Methods:**

A mixed methods case study design was adopted. Two states, Uttar Pradesh (representing a trust model) and Jharkhand (representing the insurance model) were purposively selected. Data sources included document reviews, key informant interviews, quantitative scheme data from the provider empanelment and claims database, and primary data on costs. Descriptive statistics were reported for quantitative data, content analysis was used for thematic reporting of qualitative data.

**Results:**

In both models, the state was the final authority on empanelment decisions, with no significant influence of the insurance company. Private hospitals constituted the majority of empanelled providers, with wide variations in district-wise distribution of bed capacities in both states. The urgency of completing empanelment in the early days of the scheme created the need for both states to re-review hospitals and de-empanel those not meeting requirements. Very few quality- accredited private hospitals were empaneled. The trust displayed more oversight of support agencies for claim management, longer processing times, a higher claim rejection rate and numbers of queries raised, as compared to the insurance model. Support agencies in both states faced challenges in assessing the clinical decisions of hospitals. Cost-effectiveness showed mixed results; the trust cost less than the insurance model per beneficiary enrolled, but more per claim generated.

**Conclusions:**

Efforts are required to enable a better distribution and ensure quality of care in empanelled hospitals. The adoption of standard treatment guidelines is needed to support hospitals and implementing agencies in better claim management. The oversight of agencies through enforcement of contracts remains vital in both models. Assessing the comparative performance of trusts and insurance companies in more states at later stages of scheme implementation, would be further useful to determine their cost-effectiveness as purchasers.

**Supplementary Information:**

The online version contains supplementary material available at 10.1186/s12913-022-08407-2.

## Background

Publicly financed health insurance schemes have been adopted by several low- and middle-income countries (LMICs) including India over the last decade, as one of the means of overcoming the challenges of the limited supply of publicly provided services and moving towards the goal of Universal Health Coverage (UHC) [1–3].

The Ayushman Bharat- Pradhan Mantri Jan Arogya Yojana (PM-JAY), is India’s most recent national-level publicly financed health insurance scheme and was launched towards the end of 2018. PM-JAY has replaced the earlier existing Rashtriya Swasthya Bima Yojana (RSBY) and aims to expand financial protection to approximately 7000 USD per year per family, consequently also expanding the benefits package to include more secondary and tertiary treatments. The scheme also removed the earlier limit on the number of family members who could avail the benefits of the scheme and claims an eligible population of about 500 million beneficiaries from the lowest socio-economic strata [4].

In assessing the effectiveness of RSBY and other earlier existing publicly financed health insurance schemes, mixed findings have been reported. Outcomes such as access to care, as measured through hospitalization rates, appear to have improved in populations covered under these schemes [5, 6]. However, the impact on financial protection as measured through out-of-pocket expenditure and catastrophic headcounts was not significant [6–10]. Studies exploring the institutional and governance arrangements of these schemes indicate challenges that may have limited the achievement of scheme objectives [11–14]. Several of these challenges relate to the institutional arrangements for healthcare purchasing adopted in these schemes.

The trust and insurance models are two alternative institutional arrangements for healthcare purchasing that have been adopted in the publicly financed health insurance schemes in India, including PMJAY. In the trust model, the public payer, termed the State Health Agency (SHA) is registered as a not-for-profit trust and purchases services directly from empanelled providers. Implementation support agencies (ISAs) may be contracted to support the scheme administration functions of the trust [4]. In the insurance model, the state health agency can contract an insurance company to insure beneficiaries and pay providers for the pre-defined list of services, in return for a fixed premium per beneficiary family unit covered. Here, insurers are responsible for authorizing treatments, processing claims and paying providers. Consequently, fraud detection and overall financial risk management are undertaken by the insurance company. Insurance companies may also further contract third party administrators (TPAs) to support their functions.

Studies on earlier schemes have indicated that when insurance company profits are linked to claim pay-outs, there are incentives to unnecessarily reject claims or empanel a limited number of hospitals, or hospitals with insufficient capacities to provide services [12–14]. Such misalignments of incentives between insurance companies and the government, suggest that healthcare purchasing through an insurance company may limit the achievement of programme objectives that seek to expand access. On the other hand, arguments have also been made about the inefficiencies of government functionaries in administering large financing schemes within a trust, due to the lack of required expertise, inability to control supplier-induced demand, or to absorb risk [13, 15], as also the fixed costs of setting up the state health agency.

The for-profit motives of private sector insurance companies to minimize claims payouts and maximize profit, are in conflict with the objectives of such schemes, which were to increase healthcare access and financial protection for the beneficiary population [11, 14]. In analyzing the contractual arrangements between the public payer (central and state governments) and private entities (insurers who functioned as purchasers and the healthcare providers), both, design and implementation indicated limited oversight and stewardship of the scheme by government [11, 12, 14]. Strategic purchasing frameworks emphasize the necessary stewardship of purchasing arrangements by governments, to ensure that purchasing decisions reflect public health priorities [16, 17]. Whether purchasing arrangements include only state entities, or a mix of public and private agencies contracted to increase capacities, experiences of other LMICs, as well as countries such as China and Thailand that have made rapid strides in their UHC journey, indicate that the capacities and incentives of purchasing agencies, and their oversight, can be important determinants to effective purchasing [18–21].

In the context of these observations, it is important to understand the experience of the trust and insurance models of purchasing services in the newly launched PMJAY, in which states have been provided the choice of model to be adopted for these purchasing actions. Very limited peer-reviewed literature is available on the performance of the scheme since its launch [22–25]. This study originated based on requests from the policy designers and implementers of PMJAY, to provide early insights into the experiences of states adopting either model. Based on policymaker concerns around how states with either model were performing on the empanelment and claim management functions in this scheme, in light of adverse experiences with similar schemes as described above, we focussed on these two key purchasing functions. Our objectives were; a) To provide a descriptive analysis of the institutional agencies for provider contracting and claim management in either model b) To assess performance on provider contracting c) To assess performance on claim management d) To understand implementation costs; during the initial phases of implementation.

These findings would serve as guidance to policy makers on further decisions regarding purchasing functions. The study also adds to the existing literature on the experiences of LMICs with different purchasing arrangements under health financing schemes, targeted at large sections of the population.

## Methods

### Study design and setting

We adopted a case study approach using mixed methods. Uttar Pradesh (UP, total population = 200 million, implementing the scheme in trust mode) [26] and Jharkhand (JH, total population = 33 million, implementing the scheme in mixed mode with a predominantly insurance component) [27] were selected purposively in consultation with the National Health Authority (apex body responsible for scheme implementation) as examples of states adopting different implementation models. For conducting interviews, we selected eight districts within Uttar Pradesh and four districts within Jharkhand, considering geographical and urban/rural mix.

### Data collection

In this mixed methods approach, we used a combination of qualitative and quantitative data for analysis.

#### Qualitative data

Qualitative data consisted of scheme-related documents and in-depth interviews with implementing agencies. For the former, we collated national guidelines and state level documentation on institutional structures and implementation processes, which included government orders, contracts with partner agencies and meeting records (see Supplementary Table 1, Additional File 1 for the list of documents reviewed). Based on the review of documents, exploratory and in-depth interviews were conducted with key stakeholders from the state health agencies, implementation support agencies, third-party administrators, insurance company and empanelled health care providers (EHCPs); using a set of guiding questions and a semi-structured format. Additional in-depth interviews were conducted subsequently with stakeholders at the state and district level, including District Implementation Unit (DIU) offices, district teams of implementation support agencies, district coordinators of third-party administrators, hospital administrators/ owners and ‘ayushman mitras’ (AM), who were contracted staff based in empanelled hospitals and co-ordinated PMJAY related functions for the hospital. In total, in Uttar Pradesh, 48 officials were interviewed over 28 interview sessions (some officials agreed to joint interviews) and in Jharkhand, we interviewed 34 officials over 30 interview sessions (see Supplementary Table 2, Additional File 1 for respondents interviewed). Informed consent was taken prior to conduct of the interviews. Interviews were recorded where consent was obtained to do so. Where interviews could not be recorded, detailed notes were prepared which were further used for coding and extraction of themes. Data was imported into NVivo12, and textual analysis was carried out using open coding. At a secondary level, patterns emerging from the text were identified and the most common pattern is reported.

#### Quantitative data

We obtained data from the Transaction Management System, Hospital Empanelment Management system and Beneficiary Identification System of the PMJAY management information systems, for the period from September 2018 to April 2019 for both states (the scheme officially commenced on 23^rd^ September 2018). These data were extracted by the National Health Authority and provided to us for the purpose of the study. For the analysis of pre-authorization and claim approvals, rejections, turn-around-times and associated variables, information from hospitals within the two states from October ‘18 to March’19 were included representing the first six months of operationalization. To enable sufficient days for claim reimbursement processes to have been completed, claims with a decision provided up to the 8th of May 2019 were included, as Uttar Pradesh had adopted a 30- day guideline for claim reimbursements, in contrast to the 15-day national guideline. A random sample of 800 claims (400 from each state) was extracted from the claim dataset, in order to carry out the analysis of the number of queries. Implementation cost data was collected from state health agencies though structured formats. Annual cost data was collected for the first year of the scheme starting at the end of September 2018, and cost-effectiveness estimations were calculated for the first six months up to March 2019. The data was processed, and initial analysis carried out in MS Excel, with further analysis undertaken using SPSS v.21.

### Analysis

The qualitative and quantitative data were analysed complementarily and our results are presented along the following themes, as described below. We describe the methodological approach for each of our objectives.

- Descriptive analysis of institutional agencies: This objective was aimed at exploring the questions below:
  i. What were the structures of the trust and insurance model, as had been set up in Uttar Pradesh and Jharkhand respectively?
  ii. What was the distribution of roles among agencies constituting each model, and specifically for the provider contracting and claim management functions?
  These questions were analysed qualitatively, through an in-depth review of scheme documentation and the interviews with implementing agencies.
- Assessment of provider contracting: In order to understand the development of a provider network to serve eligible beneficiaries under the scheme, we explored this objective through a combination of quantitative and qualitative data. We explored the following questions to comprehensively assess provider contracting.
  i. What were the characteristics, distribution and quality of empanelled providers in each model?
  ii. What factors contributed to these findings on empanelled providers?
  The first question was analysed quantitatively, using data sources and indicators, as summarized in Table 1. We analysed the qualitative data thematically to obtain context and insights into possible factors contributing to our quantitative findings. Key themes have been reported around processes adopted for empanelment, motivations among empanelled providers and measures adopted for ensuring quality of hospitals contracted under the scheme.
- Assessment of claim management: To assess the performance of both models on claim management, we analysed both quantitative and qualitative data. Specifically, we explored the following questions:
  i. How did the efficiencies of the two models compare on claims processing with respect to timeliness of payments to providers?
  ii. What factors contributed to these findings?
  iii. How did claim rejection rates compare in the two models?
  iv. What were the challenges experienced in the claim auditing process?
  We analysed the first three questions mainly through quantitative data, supported by qualitative insights to corroborate our findings, and provide context and generate emergent themes such as the challenges experienced in claim auditing processes. Claim management efficiency was assessed through turn-around times (TAT) of claim settlement and pre-authorization decisions. TAT for claim settlement was defined as the number of days taken from the time a claim was submitted by a hospital on the transaction management system, to its final resolution, which could be the payment against the claim or rejection of the claim. TAT for pre-authorization decisions was considered as the number of hours from the time a hospital generates a pre-authorization request for a treatment package on the transaction management system, to the time it is approved or rejected. To obtain insights into factors contributing to payment timelines and delays, we reported the association of factors including, the number of queries raised on claims, hospital sector (public and private) and the value of claims, with the delays in claim payment. Claim rejection rates were the percentage of total number of claims submitted by empanelled hospitals, that were rejected for payment. Further details on the quantitative indicators and analysis methods are summarized in Table 1.
- Implementation costs: This objective was analysed quantitatively. The cost borne by government on the scheme has two parts – the first is the total claim amount reimbursed to empanelled hospitals and the second is the administrative cost. The cost incurred on reimbursing claims is directly linked to case-based treatment package rates, which were standard in both states, and a factor of the volume of claims and type of packages claimed. Administrative costs, on the other hand, are not standard between the two models, and are also affected by the amount of work performed. On implementation costs, we therefore explored:
  i. What were the annual administrative costs in either state?
  ii. What was the administrative cost- efficiency in the first six months?

**Table 1:** Quantitative analysis methods

Area of assessment	Theme	Indicators and variables	Analysis method	Data source
Provider contracting	Characteristics of empanelled hospitals	Proportion of hospitals by: •Sector- public, private (for-profit and not-for profit) •Size- small (< 30 beds), medium (30–100 beds), large (> 100 beds) •Specialties empanelled	Descriptive statistics	Hospital Empanelment Management (HEM) system
	Distribution of empanelled hospitals	•District-wise beneficiary to bed ratio:the number of eligible beneficiaries per empanelled private hospital bed		HEM
	Quality of empanelled providers	Private hospitals by accreditation status		HEM
Claim management	Claim management efficiency	•TAT for claim settlement^a^ •TAT for pre-authorization decisions^a^	Descriptive statistics including median times and proportionate delays. Delays defined as reported TAT exceeding state guidelines	Transaction Management System (TMS)
	Factors associated with turn-around times, delays	Number of queries	Logistic regression and odds ratios. Delayed payments converted into binary categorical dependent variable	TMS sample data
		Hospital sector (public or private)	Descriptive statistics with proportionate delays	TMS and HEM
		Monetary value of claims	Correlation analysis using Spearman’s Rho correlation	TMS
	Claim rejection	•Claim rejection rates •Pre-authorization rejection rates	Descriptive statistics; overall and by hospital sector	TMS
Implementation costs	Administrative costs	Unit administrative costs (per eligible beneficiary family)	Unit annual estimated cost comparison for first year of the scheme beginning end September 2018	Primary data on costs from state health agencies; TMS and Beneficiary Identification System data for claims and beneficiary numbers
	Administrative cost effectiveness	•Administrative cost per claim •Administrative cost per beneficiary enrolled	Cost effectiveness comparisons for first six months of scheme up to March 2019	

^a^TAT calculations as per service contracts for the purpose of scheme implementation do not include the time that hospitals take to respond to queries. However, for the purpose of the study, and as per the data made available to us, TAT calculations include the total time taken from start to end of the process. These findings are indicative of efficiency of the stakeholders involved in the process

Administrative costs heads included staff salaries, transportation, materials and miscellaneous costs, and the fees paid to contracted support agencies for one year since the start of the scheme. In the insurance model, administrative costs retained by the insurance company are linked to claims ratios achieved as per the contract terms, and unspent balances are to be returned to the state health agency. Claim ratios were defined as the total claim pay-out expressed as a percentage of the total premium earned by the insurance company. For a claim ratio of < 60%, up to 10% of the premium may be retained by the insurer as administrative cost, a figure that rises to 12% for a claim ratio of 60–70%, and 15% for a claim ratio of 70–85%. Based on the claim ratio achieved by Jharkhand in six months, we estimated the annual administrative cost as 10% of the premium, which was the most conservative estimate. Cost- effectiveness was measured using claims submitted and beneficiaries enrolled as the outputs.

## Results

### Overview of institutional agencies in the two models and distribution of roles in relation to provider contracting and claim management

Uttar Pradesh adopted the trust model, while Jharkhand adopted an insurance model for the major proportion of claims generated. A brief overview of the agencies in each state model and the distribution of key purchasing functions among them, is described below. Table 2 summarizes the variations in the distribution of roles among agencies in the two state models, with respect to provider empanelment and claim management. While the processes are largely similar, the distribution of roles vary. Under the trust mode, the state health agency is the final authority for claim reimbursements; whereas in the insurance mode, the insurance company is the authority on claim decisions, with oversight by the state health agency. In terms of provider contracting, state and district empanelment committees carry out similar processes in both models, however, these committees include a representative of the insurance company in Jharkhand.

**Table 2:** Distribution of roles among agencies involved in empanelment and claim management in the two models

Function	Trust (Uttar Pradesh)	Insurance (Jharkhand)
Provider contracting	Final approvals provided by the state health agency through district and state empanelment committees	Final approvals provided by the state health agency through district and state empanelment committees. These committees include a representative of the insurance company, among their members
Claim processing	By implementation support agency, with verification and final decision on claims by the state health agency	By third-party administrators contracted by the insurance company, with random verification of a sample claims by the insurance company. The state health agency only audits rejected claims and a sample of approved claims
Reimbursement of claims to providers	By the state health agency	By the insurance company. State health agency pays the premium to the insurance company

#### State agencies and distribution of roles

In Uttar Pradesh, an existing trust formed for the implementation of the earlier RSBY scheme, was expanded in capacity to act as the state health agency for PMJAY implementation. It was headed by a Chief Executive Officer appointed from the Indian Administrative Services and supported by senior public sector medical officers, leading various scheme functions. Despite the deputation of additional staff, the state health agency was short of the planned number of human resources during the early days of scheme implementation (31 staff working out of 55 resources initially planned for recruitment). Consequently, the available staff were found to be performing multiple functions related to scheme implementation. State health agency representatives formed a part of the state empanelment committees (SEC), the final authority on provider contracting. The state health agency was also responsible for auditing claims and reimbursing providers.

Contracted resources in the trust model in Uttar Pradesh included four implementation support agencies, one agency providing technical support for fraud detection, and technical resource persons positioned within the state health agency through development partners, who provided support on multiple functions, including claim auditing. Implementation support agencies worked to support state health agencies in processing claims, and other functions. They were paid a fixed fee per eligible beneficiary family and were monitored regularly by the state health agency, through performance measures. The implementation support agencies also set up state and district-level offices as per the contract terms and were required to provide technical support to empaneled providers.

In the insurance model of Jharkhand, unlike Uttar Pradesh, the state health agency was newly constituted for PMJAY. With only five staff appointed out of 31 recommended in Jharkhand, the staff shortage was even more pronounced compared to Uttar Pradesh. Most of the key functions such as processing empanelment of providers, reviewing doubtful claims and monitoring third-party administrators, were handled by a single medical officer, who also conducted medical reviews, when required. Other staff included an accounts manager, a manager of the management information systems, and a manager for awareness and communication. The state empanelment committee in Jharkhand was different from that of the trust, in that it included a representative of the insurance company.

Jharkhand contracted a public sector insurance company, who was paid a fixed premium per beneficiary family covered, and in turn reimbursed empaneled providers for their services, at fixed case-based payment rates (the provider payment methods and rates were uniform in both states). The insurance company in turn, sub-contracted three third-party administrators, who carried out administrative functions related to claims processing, among others. The insurance company was selected by the state health agency based on a bidding process. At the time of our study, as per contracts, the insurance company could retain a percentage of the premium as administrative costs which was linked to the annual claim ratio achieved (10%, 12% and 15% for claim ratios of 60%, 60–70% and 70–85% respectively). The remaining unspent premium amounts would have to be returned to the SHA. If the claim ratio exceeded 110% and went up to 150%, the state health agency would pay 50% of the excess amount paid in claims. Beyond 150%, the insurer would have to bear all the losses. As against Uttar Pradesh, where the fee paid to implementation support agencies are fixed and not dependent on outputs, in Jharkhand, insurance companies have three levels of administrative fees that they can retain, as well as risk. At a claim ratio between 70 to 85%, the insurance company can retain the maximum proportion of the premium earned as its administrative fee. At claim ratios upward of 100%, insurance companies would have to bear financial losses. The implications of these payment terms with implementation support agencies and insurance companies are discussed at the end, in relation to our findings on claim volumes and management.

#### District agencies and distribution of roles

At the district level, the institutional structure comprised a district implementation unit (DIU) and the district resources of the implementation support agencies, third-party administrators and district committees. District implementation units and implementation support agency offices in the district supported functions of the state functionaries within districts. They coordinated across stakeholders, i.e. patients, providers and the payer, for scheme operations. With respect to provider contracting and management, DIU staff frequently approached hospitals to encourage them to be empanelled under the scheme, and visited empaneled hospitals to maintain oversight. DIUs also intervened to assist beneficiaries in approaching the appropriate hospitals for the treatment required. To assist in claim processing by the state health agency, DIU staff sometimes visited hospitals to verify beneficiary treatment details in person. In Uttar Pradesh, each DIU consisted of a nodal officer at the head, who was usually an existing health service officer given the additional charge of the PMJAY scheme oversight in the district. The DIU also comprised three contracted staff including a programme coordinator, information systems officer and grievance manager, each paid a fixed monthly salary. The DIUs in Jharkhand were not formed at the time of conducting this study, although they were a part of the proposed framework of implementation in the state and a notice to all districts had been issued by the state health agency for their formation. The operations of PMJAY at the district level were largely overseen by the civil surgeon of the district, who is the head of medical services in a given district. In addition, one member of the third-party agency was found assisting the overall implementation at the district level. There were also district empanelment committees in both states to conduct field verification of hospitals and report these to the state empanelment committee.

### Provider contracting

In this section, we begin with a brief overview of the processes adopted for empanelling hospitals under the scheme. We further report our findings on the characteristics, distribution and quality of empanelled hospitals, and associated factors.

#### Hospital empanelment processes

Healthcare providers from the public and private sectors fulfilling the empanelment criteria, are contracted to provide health services under PMJAY. In both models, empanelment processes were carried out by the empanelment committees formed at the state and district level. The process for empanelment was similar in both the models, with the exception that in Jharkhand, the contracted insurance company and third- party administrator were a part of the empanelment committees, however this was not the case in Uttar Pradesh. We found that the accountability for provider contracting in both the states was mainly held by the state health agency, with the insurance company not having much of a say in the choice of providers, to be empaneled under the scheme.

The national guidelines outline the basic and clinical specialty-specific criteria that hospitals seeking empanelment under the scheme must meet. The mandated basic criteria apply uniformly to all hospitals, whereas specialty-specific criteria intended for tertiary care specialties have the flexibility to be adapted, keeping local circumstances in mind. Both of the studied states were found to have adopted the national criteria with no state-specific modifications. Following a hospital's application for empanelment on the information management system, district committees conduct a field verification and report their findings to the state committee, which grants approval. For public hospitals, all those with in-patient care facilities were ‘deemed empaneled,' and did not have to go through any formal empanelment process. Detailed data on public hospital infrastructure was also unavailable at the time. This indicated that a proper assessment of the adequacy and readiness of public hospitals to provide services as required by the scheme, was not carried out.

#### Characteristics and motivations of empanelled hospitals

It was observed that both states had a higher proportion of private hospitals than public hospitals (75% in Uttar Pradesh and 65% in Jharkhand), among all empaneled providers **(**Table 3**).** In the absence of reliable data on available healthcare providers in the states, we were unable to ascertain the proportion of private healthcare providers who were empaneled under the scheme, in each state. The majority of empaneled private hospitals in Jharkhand (50%) were small, with less than 30 beds, while the majority in Uttar Pradesh were medium-sized hospitals, with 30 to 100 beds (47.9%).

**Table 3:** Indicators of empaneled providers

	**Uttar Pradesh**	**Jharkhand**
Total eligible beneficiary families	11,807,068	5,705,502
Total eligible beneficiaries	59,035,340	28,527,510
Number of hospitals empanelled	1724	615
Private: public hospital ratio	3:1	1.8:1
Private for-profit: private not-for-profit ratio	4.4:1	6.3:1
Number of beds empanelled^a^	77,393	13,571
Small hospitals (< 30 beds)^a^	35%	50%
Medium-sized hospitals (30–100 beds)^a^	47.9%	40.5%
Large hospitals (> 100 beds)^a^	17%	9.1%
Median bed strength^a^	30	23
Number of National Board for Accreditation of Hospitals and Healthcare providers (NABH) accredited hospitals empanelled^a^	83 of 236	6 of 10
Average number of specialities empanelled per hospital^a^	5.43	3.65

^a^data for private hospitals

In both states, the hospital authorities stated that the expectation of higher patient volumes acted as an incentive for them to be empaneled under the scheme. Some small hospitals in Jharkhand claimed that PMJAY beneficiaries made up 70 percent to 80 percent of their patient load and that the scheme had increased their overall income. However, large hospitals with better facilities, stated that the primary motivation for empanelment was to support the needy and that they make no discernible profit from it at current reimbursement rates; while some hospitals stated that they have entered the scheme out of fear of losing patients to competing facilities. These statements from large hospitals indicated that they did not consider PMJAY to be financially rewarding at the time. Despite these different responses from smaller hospitals versus larger ones, private hospitals had been keen to be empanelled under PMJAY in the early days of the scheme. The state health agency authorities agreed with these rationales as they regularly interacted with empaneled hospitals.

*“Suppose there are two hospitals and say one gets empaneled, everyone will come to him. No one will go to this other person. This way it’s a business competition. To stay in the game, they would have to get empaneled, or they would be left out” SHA 01, Jharkhand.*

*“Volumes have definitely increased because those 52 patients which we have operated, I don't think they would have got operated in private sector or general… We were expecting much more, and gradually number increased over and I think that volume will increase further..” Private hospital administrator 03, Uttar Pradesh.*

*“Package rates are very low. Medicine package rates are really low. The rates for non-Ayushman procedures are double of the Ayushman ones. We are just helping the nation.” Private hospital administrator 01, Jharkhand.*

#### Distribution of empaneled hospitals

**Fig. 1:**
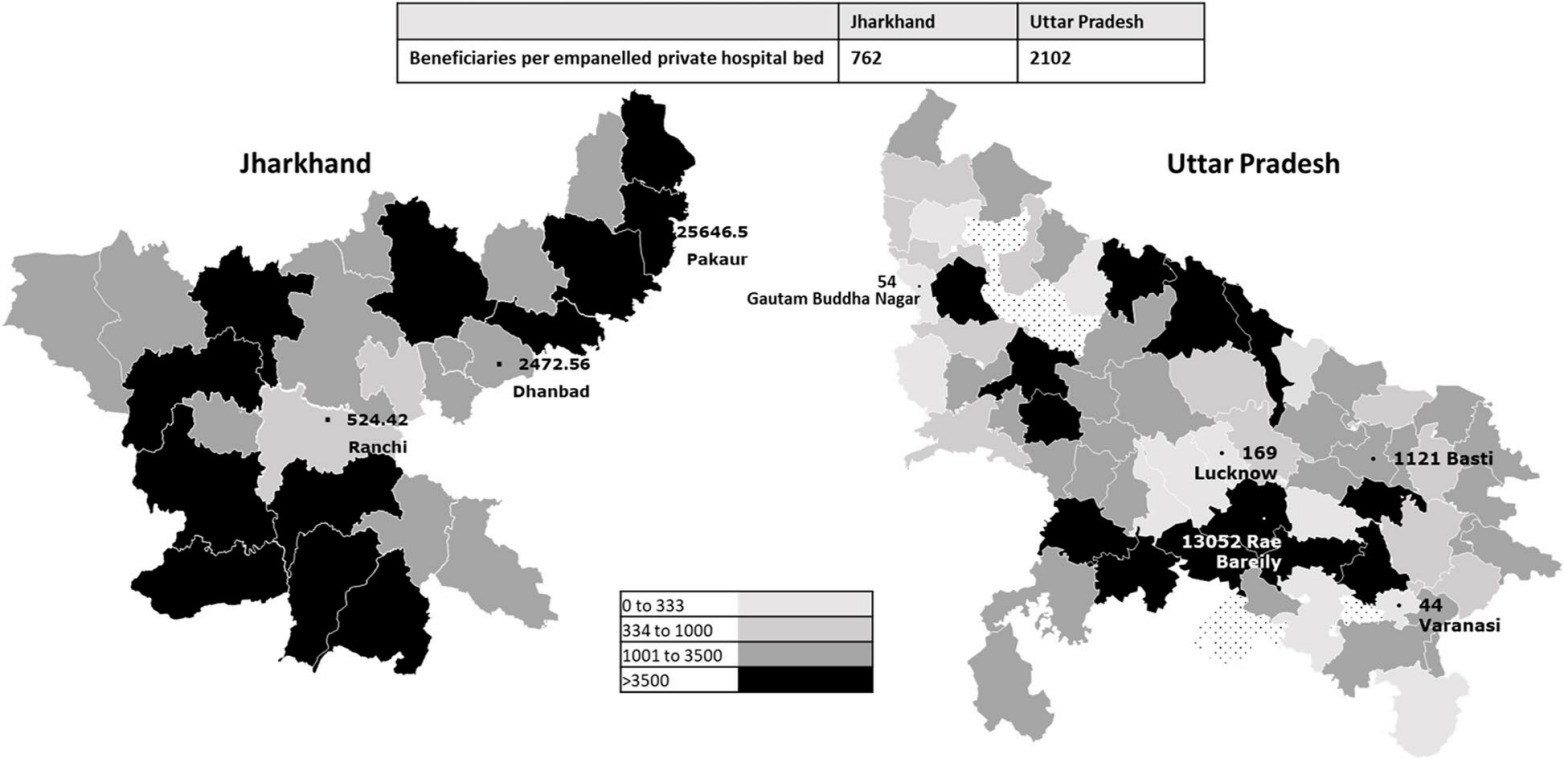
District wise distribution of empanelled hospital beds. Maps created by authors using Gramener application tools (https://gramener.com/map/ ). Maps are not to scale. Textured patterns indicate missing data for six districts in Uttar Pradesh

We used the beneficiary to bed ratio to assess the distribution of empanelled private hospitals across the states. The availability of hospital beds under the scheme varied greatly in both states. Uttar Pradesh reported an overall beneficiary-to-bed ratio of 762 beneficiaries per bed, however the distribution was uneven, with some districts reporting a much better availability of beds than others. **(**Fig. 1**).** Similarly, Jharkhand reported 2102 beneficiaries per bed and a higher proportion of districts with very low bed densities.

#### Accreditation and quality of hospitals

As per national guidelines, both states offered financial incentives to hospitals accredited by the National Board for Accreditation of Hospitals and Healthcare providers (NABH), a recognized accreditation system for healthcare providers in India. In Jharkhand, six of the ten NABH hospitals were empaneled under the scheme. In Uttar Pradesh, 35% of NABH certified hospitals were empaneled, showing a significant disparity in the scheme's involvement of hospitals with higher standards of quality certification **(**Table 3**)**.

Both states conducted the empanelment process for hospitals for the specific purpose of PMJAY implementation, including visits for inspections. Interactions with the state health agency, district officials and empanelled providers revealed that in the early days of the scheme's implementation, the goal was to empanel as many hospitals as possible and quality could not be prioritised in all cases. The state health agency in Uttar Pradesh reported they were now focussing on ensuring that appropriate hospitals remained empanelled, and new applications were being scrutinized more closely. Jharkhand announced that re-inspection of previously empanelled hospitals had begun. They also stated that approximately 20 hospitals that were empanelled in the first three months were later de-empanelled, after it was discovered that they did not meet the criteria.

### Claim management

In this section on claim management, we begin by outlining the salient features and differences between pre-authorization and claim management processes in the two models, and the distribution of roles among agencies in Table 4. We then highlight our main observations on i) Timelines of claim processing and provider payments ii) Reasons for delays in claim reimbursements iii) Claim rejection rates iv) Challenges in auditing claims.

**Table 4:** Key variations in pre-authorization and claim management processes in the two states

	**Uttar Pradesh (Trust)**	**Jharkhand (Insurance)**
Pre-authorization	Some packages are pre-approved, the remaining are to be approved by the implementation support agency. Unspecified packages are approved upon inspection by the medical management team in the state health agency	Some packages are pre-approved, the remaining, including unspecified packages, are to be approved upon inspection by the medical doctor of the third-party administrator
Claims Audits	Initial auditing of claims generated by the providers is done by the implementation support agency, and is later verified by the medical management team of the state health agency	Claims were approved by the third-party administrator, while the insurance company and state health agency only conducted audits of a random sample of claims
Claims Rejection	All final decisions on claims were provided by the state health agency, including rejections	Claims rejected by the third-party administrator were reviewed by the state health agency and the decisions would be reversed where deemed appropriate
Turn-Around Time (TAT) for Claims^a^	30 days	15 days
TAT for pre-authorizations ^b^	6 h	6 h

^a^National guideline is 15 days

^b^National guideline is 6 h

#### Turn-around time (TAT) efficiency in processing claims and pre-authorizations

The states used turn-around-times (TAT) to monitor claim management processes. We found that in terms of pre-authorizing treatments within the stipulated time, both models reported delays in a significant proportion of cases. A higher proportion of delays were seen in Jharkhand than in Uttar Pradesh (45.7% vs. 33.7%), however, both states reported a median TAT that was within the state-adopted guideline **(**Table 5**)**. Pre-authorizations that remain un-approved and with no pending queries for over six hours, get auto approved by the online system. It was also observed that of those cases exceeding the six -hour limit, 81.6% were auto-approved by the system in Jharkhand, whereas only 29% were auto-approved in Uttar Pradesh. This may be indicative of the higher volume of pre-authorization requests, or a lower efficiency in timely approvals. Although the specific reasons for this difference could not be ascertained, the majority of the cases in Jharkhand, seemed to have been eventually auto-approved by the system, which is not desirable.

**Table 5:** Timeliness of processing provider payments

Pre-authorization	Uttar Pradesh (*n* = 27,068)	Jharkhand (*n *= 46,011)
Median TAT (all cases requiring approval)	3 h 15 min	5 h 5 min
Percentage cases in which TAT exceeds 6 h	33.7%	45.7%
Cases exceeding 6 h that are system approved	29.4%	81.6%
**Claim Processing**	**Uttar Pradesh** (*n *= 44,931)	**Jharkhand** (*n* = 91,780)
Median overall TAT	32 days	15 days
Proportion of claims wherein overall TAT exceeds the state guideline	52.5%	44.7%

A reverse trend was observed in claim payments, where a higher proportion were delayed in Uttar Pradesh than in Jharkhand (52.5% vs. 44.7%). Both states, however, reported a median TAT within the state guidelines in place at the time of conducting the study, with Uttar Pradesh exceeding the guideline by two days. The contracts with the implementation support agencies and insurance company contain provisions for penalties in the case of delayed processing of pre-authorizations and claims. Providers were liable to receive their payments with interest if delayed beyond the norm. Pre-authorization delays result in a penalty per request delayed. These are significant financial amounts; however, we did not find that these had been levied on the agencies within the first six months of scheme implementation.

#### Factors associated with delayed claim payments

##### Queries

Interviews with empanelled hospitals indicated that the two states differed in their perception of time taken for payment. While the hospitals in Jharkhand generally expressed their satisfaction with timely payment, in Uttar Pradesh, the hospitals reported that payments took very long to be received. This was in line with the difference in state adopted guidelines for processing payments (15 days in Jharkhand versus 30 days in Uttar Pradesh). In the opinion of some third-party administrator managers and hospitals, many hospitals have shown interest in obtaining empanelment after witnessing the timely payment being made to other hospitals. Providers in Uttar Pradesh appeared to be more troubled by the high number of queries raised on claims and repetitive nature of the queries. Public sector providers faced a manpower shortage, despite ayushman mitras being positioned in the hospitals. The high patient volumes treated there generated a significant workload and available manpower appeared to be insufficient to manage the necessary documentation required for processing claims. They expressed their dissatisfaction with the large number of queries, which delayed processes further.

“…first of all, queries are a big problem; and secondly, payments are very slow.” Private hospital administrator 02, Uttar Pradesh.

“…the number of queries is so large, that by the time they are answered, and they reach the Claims Processing Doctor (CPD), about one and a half months go by.” Private hospital administrator 05, Uttar Pradesh.

We also found that Uttar Pradesh raised a higher number of queries ranging up to six per claim, whereas in Jharkhand, a maximum of four queries per claim were raised (*n *= 400 for each state). Queries largely consisted of requests for supporting documentation for the clinical procedures undertaken at the hospital. Delayed claim settlements were predicted to a larger extent by the number of queries in Jharkhand, as compared to Uttar Pradesh (OR = 12.5 in Jharkhand; 2.3 in Uttar Pradesh, *p* < 0.01). This observation was likely due to the double auditing process undertaken by the implementation support agency and state health agency at Uttar Pradesh, in the early months of the scheme, which mainly determined timelines for claim reimbursements.

##### Type of hospitals and value of claims

We also analysed the association between delayed claim payments and the hospital sector (public vs. private), and claim value.

We found that in Uttar Pradesh, 55% of the claims from private hospitals (*n* = 35,909) were settled late, whereas 42.2% of claims from public hospitals (*n *= 8968) were delayed for reimbursement. In Jharkhand, a similar but smaller difference was observed in delayed claim settlement between private (45.4%, *n* = 78,008) and public hospitals (41%, *n* = 13,575).

We hypothesized that higher value treatment packages (higher claim amounts) would take longer to process, as more stringency would be applied to these claims. We therefore tested the correlation between these two variables, i.e. claim turn-around-time and value of treatment/claim and found no correlation for neither Uttar Pradesh (*r* = 0.076, *p* < 0.01, *n* = 44,931) nor Jharkhand (*r* = 0.068, *p* < 0.01, *n *= 91,780). We therefore concluded that claim values did not necessarily influence the time taken to audit claims and provide a decision.

#### Claim rejection rates

Claim rejection rates were calculated for both the states, as the percentage of total number of claims submitted by empanelled hospitals, that were rejected for payment within the study period. Claim rejection rates were found to be higher in Uttar Pradesh (5.5%, *n* = 59,468) than in Jharkhand (1.2%, *n* = 100,260). The pre-authorization rejection rate was also higher in Uttar Pradesh than in Jharkhand (2.3%, *n* = 89,075 vs. 0.7%, *n* = 138,874). These findings were contrary to expectations and are further elucidated in the discussion. In terms of the type of hospital, the rejection rate in Uttar Pradesh was higher for claims generated by private hospitals as compared to those generated in public hospitals (6% vs. 3.4%). In Jharkhand, the claim rejection rates for private and public hospitals were the same (1.2%). The reasons for this observation merit further study.

#### Challenges in claims auditing

In order to monitor the quality of claim auditing carried out, penalties were also levied by the trust in Uttar Pradesh on certain implementation support agencies, for wrongfully approved claims. During interviews, it emerged that these financial penalties were substantive, amounting to approximately 25,000 USD for about 20 to 25 claims wrongfully approved, since the start of the scheme to the time of data collection. The state health agency was thus reportedly maintaining oversight and control of  the performance of implementation support agencies. This was further explored from the point of view of the implementation support agency. It was perceived that claim audits are largely the opinion of ‘one doctor against another’. In certain cases, a treating doctor at the empanelled hospital is a specialist, more highly qualified than the implementation support agency clinician reviewing the claim. The treating doctor may deem a certain treatment necessary for a patient on the basis of his own diagnosis. The documentation requirements of the scheme for approval of such claims, however, might imply that the doctor should follow a different diagnostic algorithm. This, in a sense, questions the clinical aptitude of the treating doctor. Implementation support agencies therefore justified their actions stating that questioning the doctor’s clinical judgement was not within their rights and hence approved certain claims where this might be the case. In other instances, there appeared to be a disparity in the seniority of treating doctors and ISA clinicians appointed for approving claims.

*“The problem of line of treatment could occur, for instance, a stent is placed in a patient, but the investigations done and documented do not prove that the patient had had a blockage or not and indeed needed a stent; or they just did the procedure to make money from the government. So, in such cases we may ask the treating doctor. But this can only be done by a qualified doctor, else the hospital can take action against us.” Implementation support agency official 02, Uttar Pradesh.*

The similar challenge was also reported by the third-party administrators in Jharkhand. The lack of specialist doctors within ISA teams, and the limited clinical experience of the medical graduates working in these teams, further limit their ability to confidently audit claims and manage providers.

### Implementation costs of the scheme

#### Administrative costs

In order to obtain preliminary insights into the implementation costs of the two models, we estimated administrative costs and administrative cost- effectiveness.

The total estimated annual administrative cost as reported in Uttar Pradesh for the first year of the scheme (beginning from September 2018) amounted to 347 million Indian rupees (~ 4.8 million USD) (see Supplementary table 3, Additional File 1 for break-up of administrative costs). Administrative costs of the trust model are borne by the state health agency and constitute salaries of the state health agency and district implementation unit staff, as well as the transportation, materials and miscellaneous budget lines for these agencies. Further, administrative costs include the value of the contracts with outsourced agencies, i.e. the agency for fraud management, and all four implementation support agencies. The data on human resources was limited in that the salaries of regular government employees of the district units (nodal officers who were given additional charge of PMJAY) was not included in these estimates. The annual administrative cost in the insurance model of Jharkhand amounted to 513.5 million Indian rupees (~ 7.1 million USD). This included the administrative costs retained by the insurance company. The quantum of administrative costs that can be retained by the insurance company is linked to the claims ratio that is achieved in the year, as per contract terms. For our study period covered, i.e. six months of the scheme from September 2018 up to March 2019, the claims ratio in Jharkhand’s insurance model was 32.4% (Table 6). Jharkhand had a small state health agency and had not set up district units, the state therefore did not provide separate administrative costs of the state health agency. The costs for Jharkhand are therefore an under-estimate of its actual implementation costs. Despite this, the unit administrative cost (per beneficiary family unit), was found to be three times higher in the insurance model of Jharkhand than in the trust model of Uttar Pradesh.

**Table 6:** Administrative cost-effectiveness in the two states (September 2018 to March 2019)^a^

	**Uttar Pradesh**	**Jharkhand**
Total eligible beneficiary families^b^	11,807,068	5,705,502
Total number of beneficiaries enrolled (% of eligible) (up to March 2019)	2,801,691 (4.7%)	3,093,659 (10.8%)
Total annual premium	-	513,495,180
Utilization rate in 6 months (claims per 1000 eligible beneficiaries) (up to march 2019)	1 per 1000 eligible beneficiaries	3.5 per 1000 eligible beneficiaries
Number of claims generated in 6 months (up to march 2019)	59,468	100,260
Total claim pay-out in 6 months (up to march 2019)	453,843,762 INR (6.3 million USD)	830,542,833 INR (11.5 million USD)
Claims ratio (6 months) (up to march 2019)	-	32.4%
Total annual administrative cost ^b^	347,004,095.32 INR (4.8 million USD)	513,495,180 INR (7.1 million USD)
Administrative cost estimated for six months (up to march 2019)	173,502,047.7 INR (2.4 million USD)	256,747,590 INR (3.6 million USD)
Administrative cost per eligible beneficiary family unit (annual)	29.4 INR (0.4 USD)	90 INR (1.2 USD)
Administrative cost per beneficiary enrolled (up to March 2019)	61.9 INR (0.9 USD)	83 INR (1.1 USD)
Administrative cost per claim submitted (up to March 2019)	2,917 INR (40.4 USD)	2,560 INR (35.4 USD)

^a^Scheme officially commenced on 23^rd^ September, 2018 (nearing the end of the month)

^b^Data provided by the state health agencies of Uttar Pradesh and Jharkhand as of July 2019

*INR* Indian rupees, *USD* United States dollars

#### Administrative cost- effectiveness

In order to estimate administrative cost-effectiveness, we used the number of claims processed and the number of beneficiaries enrolled as the two outputs. We estimated administrative cost efficiency for the first six months of the scheme, up to March 2019 (the scheme officially commenced on 23^rd^ September, 2018). During the first six months of the scheme, Jharkhand reported a higher number of claims generated (100,260) as compared to Uttar Pradesh (59,468), despite having half as many eligible beneficiary families; thereby reporting a higher utilization of the scheme. Although utilization of the scheme was entitlement-based and prior enrolment was not necessary, we observed that Jharkhand also had enrolled a higher proportion of beneficiaries than Uttar Pradesh.**(**Table 6**).**

In terms of cost-effectiveness, the administrative cost of the scheme per enrolled beneficiary was 34% higher in Jharkhand than in Uttar Pradesh. However, the cost per claim was lower in Jharkhand than in Uttar Pradesh, by about 14%. This reflected the higher utilization levels achieved by Jharkhand at the time of the study. Therefore, while insurance unit administrative costs were higher than that of the trust, cost-effectiveness showed mixed results. In terms of claims generated, Jharkhand reported a better cost-effectiveness for the study period. Due to the early days of scheme implementation, these costs and outputs would have to be monitored closely, as the scheme progresses and stabilizes.

## Discussion

In this study, we aimed to provide insights into the trust and insurance models of healthcare purchasing in PMJAY, with respect to provider contracting, claim management and implementation costs, during the early phase of the scheme. We discuss here our main findings and offer some recommendations for policy and further research.

Our first main observation was that irrespective of the model adopted, many contracted resources form a part of the institutional structures implementing the scheme at the state and district levels. Key functions such as processing treatment pre-authorization requests and processing provider claims were carried out by insurance companies, implementation support agencies and resources provided to the government through development partners organizations, as technical support for these operations. While such arrangements provide the much-needed additional capacity for implementing large schemes, they also cause concerns about institution building, and the ability of the system to develop and retain the expertise required for sustaining operations of such financing schemes. Long-term partnerships and continuous oversight by the state health agency, would be required to continue with such arrangements.

One of the key elements of health protection schemes is the network of providers empanelled within the scheme. Under PMJAY, we did not find any significant differences in the criteria and processes for contracting providers adopted by the states with either model. A key observation with respect to provider contracting was that irrespective of the model, the State was the decision-making authority on contracting hospitals. This was in contrast to the erstwhile RSBY, where the insurance company was primarily responsible for empanelment.

We also observed that a fresh round of inspections had been conducted in these two states for contracting providers, rather than continuing with the RSBY empaneled hospitals. However, public hospitals were deemed empanelled, and this was likely to impact their readiness and ability to provide services under the scheme. Data on infrastructure and specialties of public hospitals had also not been maintained in the empanelment database. While good processes had been put into place for contracting private hospitals, there were some limitations. Due to the urgent need to empanel sufficient hospitals in a very little amount of time at the start of the scheme, some compromises might have been made on the quality of these hospitals. However, states were in the process of reviewing empanelled hospitals, to ensure that they continued to meet the necessary criteria and some had been consequently de-empaneled. This process needs to be periodically carried out to ensure adequate oversight of hospitals, by state health agencies.

Consequently, the observed variations in distribution and characteristics of empanelled private hospitals appeared to reflect the available infrastructure in the two states. Uttar Pradesh had a higher density of empaneled hospital beds per beneficiary family, as well as specialist services as compared to Jharkhand, with large variations in available bed strength across districts in both states. Although as per guidelines, states could relax some empanelment criteria for specialist services, we did not find either state had done this. More guidance to states on this aspect would be helpful, to enable them to address the skewed distribution of providers across districts. Complete data on empanelled public hospitals would serve to further assess the adequacy of empanelled hospitals in the scheme.

Both states empanelled a higher number of private hospitals, as compared to public hospitals. This possibly reflects the higher number of private hospitals estimated to be available in both states, as compared to public hospitals [28, 29]. These trends are reflected at the national level, with both models showing a similar distribution of public–private mix of hospitals [30]. Reliable data on private sector infrastructure in India is however lacking, rendering it challenging to estimate the effectiveness of the scheme in drawing participation from the large private sector that otherwise provides the major proportion of in-patient and out-patient care in India [28, 31]. Mid-sized hospitals formed a larger proportion of all empaneled hospitals in UP, as compared to Jharkhand wherein small hospitals formed half of all empaneled hospitals. In terms of quality of care, overall, very few accredited hospitals were found to be participating in the scheme. These findings, therefore, appear to highlight the available health infrastructure in the states. Both Uttar Pradesh and Jharkhand report a scarcity of specialist healthcare providers in rural areas [32]. These issues are not confined to the states we studied, but possibly highlight the persisting supply-side gaps in many states of India [29, 33].

Provider payments were processed differently in the two models due to the involvement of the insurance company in Jharkhand, versus the implementation support agencies working with the state health agency in Uttar Pradesh. The oversight of the contracted agency was found to be much higher by the state health agency in Uttar Pradesh, wherein it conducted audits of all claims that were initially processed by the implementation support agencies, and levied significant financial penalties in cases of wrongful claim approvals by the agencies. This scrutiny was further reflected in longer turn-around times for provider reimbursement and repeated queries on the claims raised. Higher claim rejection rates were also reported by the trust in Uttar Pradesh than the insurance company in Jharkhand. These observations indicate two important issues.

Firstly, the higher claim rejections, queries and oversight of the implementation support agencies by Uttar Pradesh, reflect that the trust had the capacity to be vigilant with the implementation support agencies and hospitals, and thereby prevent wrongful claim approvals. This is in contrast to earlier experiences with public–private partnerships in health that have reported instances of the inability of government bodies to monitor their partners in the private sector, including ensuring rightful implementation of the contract terms and service delivery output [12, 34]. We could not conclusively determine whether the higher vigilance was due to a cautionary approach taken by state officials, based on their earlier experiences of corruption reported under RSBY [35, 36].

However, the second possible implication was that long turn-around times, numerous queries and high rejections due to incorrect documentation for claim reimbursements, also indicated weak capacities of hospitals in complying with these processes. We did not have any evidence of differential capacities of hospitals in Uttar Pradesh and Jharkhand to conclusively determine the reasons for higher rejection rates in Uttar Pradesh, as compared to Jharkhand. However, these observations merit further study, in order to prevent these challenges for hospitals and to improve claim processing efficiencies in the state.

We also found that the claim rejection rate in Uttar Pradesh was comparable to that of the trust in the Rajiv Arogyashri scheme of Andhra Pradesh in its initial year of implementation (4%), however, the rejection rate in the Rajiv Arogyashri scheme increased subsequently up to about 10% [13, 37]. The trust in Karnataka’s publicly financed health insurance scheme reported a rejection rate of 12% [37]. These rates, as reported by trusts in other states, were however always lower than those reported by insurance companies (in the range of 16%), which was in contrast to our observations [13, 37].

Consequently, our observations on lower claim rejection rates by the insurance company in Jharkhand was unexpected since insurance companies have been previously seen to hold back payments to hospitals, as a means to minimize claim payouts, and thereby increase their profits [6, 13]. However, the contract terms between the state health agency and insurance company in PMJAY imposed a ceiling on the administrative costs that insurance companies could retain that was linked to the claim ratios achieved. Unspent balances were to be returned to the state health agency. Insurance companies were allowed to retain a maximum proportion of premiums received as administrative costs if they achieved claims ratios of 70 to 85%, which was possibly incentivizing the insurance company, in this case, to achieve claims ratios in this range.

Jharkhand reported a claims ratio of 32.4% in the first six months of the scheme. Scheme utilization was higher in Jharkhand, as compared to Uttar Pradesh, despite Uttar Pradesh having double the number of eligible beneficiary families. The implementation support agencies in Uttar Pradesh had fixed payment terms, independent of the claim volumes processed. Several factors other than the models and purchasing agencies are likely to influence the utilization of the scheme in both states. A comprehensive assessment of these factors was beyond the scope of the objectives of this study. Our findings, however, indicate that the claim approving behavior of the insurance company could have been influenced by the incentives created by the payment terms in the contract, partially affecting utilization in Jharkhand. Such practices by insurance companies have been earlier reported in Rajiv Arogyashri wherein claims ratios somehow remained at the level at which administrative fees retained, were at the highest proportion permissible (these contracts had similarly designed payment terms) [13]. However, these observations from PMJAY are early in the scheme implementation phase and would need to be repeated after the completion of one or more policy periods, in order to better understand the claim approving behaviour of the insurance company. A comprehensive assessment of the factors contributing to higher scheme utilization in Jharkhand as compared to Uttar Pradesh, also merits assessment.

Checks and balances for the prevention of supplier-induced demand are also dependent on appropriate validation of all pre-authorization and claim requests, while processing payments. For-profit providers are likely to indulge in such behavior irrespective of the type of purchaser, when the type of payment remains the same (a type of case-based payment referred to as a package rate in PMJAY) [38]. The concern over the occurrence of supplier-induced demand remains, irrespective of whether the purchaser is a private insurance company or a public agency [7, 13, 39]. While our study does not explore its occurrence, we found the lack of adequately qualified doctors on the teams of the contracted agencies processing claims in both models was likely to influence the effectiveness of processing provider payments, while preventing unnecessary treatments. This was reflected in our interviews with implementation support agencies, who expressed their inability to question treating doctors over their chosen treatment pathways. In Uttar Pradesh, the double auditing of claims by qualified doctors in the state health agency fulfilled this gap within implementation support agencies. However, national guidelines do not necessitate that state health agencies audit all claims; hence this may not be the case in other states adopting the trust model. Contracted agencies in both models, therefore, could not adequately evaluate the clinical decision-making of hospitals. These observations call for the timely uptake of standard treatment guidelines for treatment conditions available under the scheme. These guidelines would also help to improve the capacities of hospitals and prevent high claim rejections. Standard treatment guidelines have been recently notified by NHA and will have to be studied once sufficiently operationalized, with regards to their scope in supporting claim management processes [40].

These observations also reiterate the vital role of oversight of purchasing agencies by the state, in both models. Enforcing contract terms to build accountability remains important to ensure that processes are followed, and is an important aspect of  the governance of strategic purchasing in such schemes [16, 17]. We found this oversight to be better ensured in the functioning of the trust model in our case studies. However, these observations need to be further explored in a larger number of states, and at later phases of scheme implementation. Such studies will remain important as the scheme grows and expands. Experiences in Nigeria and Malawi indicate that limitations in capacities of the purchasers, as well as inadequate oversight of these agencies by the state, contributed to ineffective purchasing in their publicly financed health insurance schemes [19, 20]. Experiences with strategic purchasing in Thailand’s universal coverage scheme also report that penalties and incentives are one of the components contributing to the effectiveness of purchasing [21]. Although China has made significant progress in health financing reforms, the country still faces problems with the capacities of the third-party purchaser in ensuring cost-effective care under its social health insurance system [18].

Our study of initial implementation costs is limited in some ways, but provides useful early insights into possible implications of adopting either model in the scheme. We found the unit administrative costs in the trust model studied to be lower than in the insurance model. However, cost-effectiveness showed mixed results. Uttar Pradesh showed slightly better administrative cost-effectiveness in terms of beneficiaries enrolled, but Jharkhand reported better cost-effectiveness in terms of claims generated. These observations reflected Jharkhand’s higher utilization of the scheme, as discussed earlier. These observations exacerbate the need for a more comprehensive assessment of factors driving utilization in states, and the resulting cost-efficiencies of the models adopted. We could not compare our findings on the cost-effectiveness of the two models to others, due to differences in definitions and methods [13]. However, costs would have to be assessed again when utilization stabilizes and in a larger number of states, to determine the cost-effectiveness of either purchasing model.

Our study is limited in that one state with either model was included. The study was commissioned at an early phase of the scheme, and hence several unknown implementation factors other than those reported could have been at play, to affect outputs reported in this study. Our cost data were also limited by the availability of complete data, as well as by the fact that we used partially estimated costs, rather than all actual expenditures due to the timing of this study. However, the states included are large and populous states, that are accorded policy priority in India. Insights into their early experiences with provider empanelment, claims management and indicative implementation costs were important to inform further policy and implementation decisions on an ambitious scheme such as PMJAY. The behaviors of institutions such as trusts and insurance companies in more states, with either model, need to be assessed at later stages of scheme stabilization to further inform scheme design, going forward. Until then, in both models, the state health agencies will have to ensure that capacities are built and retained, and that the incentives for the supporting agencies are continually assessed and re-designed, if needed, to ensure that scheme objectives are achieved.

## Conclusions

Due to the large presence of outsourced agencies and technical partners involved in both models of purchasing under the scheme, measures for retaining capacity and technical expertise within these institutions must be taken, for its long-term sustainability. Provider empanelment processes were largely driven by the state, irrespective of the model adopted. Private providers formed the majority of empanelled health care providers, with largely uneven distributions across districts. Complete data on public hospitals are further required to assess the overall adequacy of the supply-side, in schemes like PMJAY. Although states adopted national guidelines with no state-specific modifications, this flexibility merits exploration to address the uneven distribution of providers. Empanelment processes had been undertaken very quickly in a limited time frame at the start of scheme implementation, calling for additional efforts to ensure the quality of empanelled hospitals is monitored. Performance on claims management was in line with payment terms and incentives built into contracts between the state and the insurance company or implementation support agencies. This indicates that the oversight of purchasing agencies by the state, including enforcement of contract terms to ensure accountability, remains vital irrespective of the model adopted. Contracted agencies in both models could not adequately evaluate the clinical decision-making of hospitals, due to differences in clinical opinions. The trust was found to display higher oversight of support agencies in claim approving processes than the insurance state in our case studies, to account for this. However, possible gaps in the capacities of hospitals to adopt claim processing guidelines were also indicated. These observations call for the timely uptake of standard treatment guidelines for treatment conditions available under the scheme. The trust reported lower unit administrative costs, but cost-efficiency showed mixed results. The trust cost less per beneficiary enrolled but the insurance model cost less per claim generated, reflecting the higher utilization in the state studied. As scheme utilization increases and stabilizes, the comparative performance of trusts and insurance companies would require to be further assessed, and in a larger number of states, to determine their cost-effectiveness as purchasers in the scheme.

## Supplementary Information

**Additional file 1.**

## Data Availability

The secondary data contained in this study were made available to the research team by the National Health Authority, India, for exclusive use in this study. Data belong to the National Health Authority. For more information please contact Ruchira Agrawal (mel.cons1@nha.gov.in) within the National Health Authority.

## Abbreviations

AM: Ayushman mitra
CPD: Claims processing doctor
DEC: District empanelment committee
DIU: District implementation unit
ECHP: Empaneled health care provider
HEM: Hospital empanelment management system
IC: Insurance company
INR: Indian rupees
ISA: Implementation support agency
JH: Jharkhand
LMIC: Low and middle- income countries
NHA: National Health Authority
OR: Odds ratio
PMJAY: Pradhan Mantri Jan Arogya Yojana
RSBY: Rashtriya Swasthya Bima Yojana
SEC: State empanelment committee
SHA: State health agency
SPSS: Statistical package for social sciences
TAT: Turn- around time
TMS: Transaction management system
TPA: Third-party agency
UHC: Universal health coverage
UP: Uttar Pradesh
USD: United States dollar
