# Supplementary material for: The trust and insurance models of healthcare purchasing in the Ayushman Bharat Pradhan Mantri Jan Arogya Yojana in India: early findings from case studies of two states
Source: BMC Health Serv Res. 2022 Aug 18;22:1056. doi: 10.1186/s12913-022-08407-2 (PMC9389741; doi:10.1186/s12913-022-08407-2)
Supplement: Supplementary file 1 — Additional file 1. [file 12913_2022_8407_MOESM1_ESM.pdf]

Supplementary Table 1. List of documents reviewed

National documents	State documents	
	**Uttar Pradesh**	**Jharkhand**
Formation of State Health Agency and District Implementation Unit under Ayushman Bharat-National Health Protection Mission<br><br>Draft Document on Structure and Tasks of State Health Agency for Implementing Pradhan Mantri Rashtriya Swashtya Suraksha Mission[a] in Trust Mode<br><br>Guidelines for hospital empanelment<br><br>Empanelment and packages<br><br>PMJAY Process flow at empanelled hospitals<br><br>Guidelines for claim settlement<br><br>Anti-fraud guidelines<br><br>Model Agreement Assurance Model with Empanelled Health Care Provider (EHCP)<br><br>Model Tender Agreement for Implementation Support Agency<br><br>Model Tender Agreement for Insurance Company	All government orders related to scheme operations issued in the public domain These were related to:<br><br>Pilot run of scheme; SHA and Governing Council formation; Formation of various State and District Committees; scheme details including benefits, number of beneficiaries, terminology; Hospital empanelment; District manpower commissioning; general orders related to training, budget allocations<br><br>Claim processing guidelines<br><br>Tender document between State Health Agency and Implementation Support Agency	Contract document between State Health Agency and Insurance Company<br><br>Governing Council Meeting Minutes

[a] Early nomenclature used for PMJAY

Supplementary Table 2. List of respondents interviewed

Stakeholder group	Jharkhand	Uttar Pradesh
**State health agency**	1 Governing council member	1 Governing council member
	1 Deputy director	1 State nodal officer
	Medical officer in-charge	2 Joint directors
	Finance manager	1 member of the medical audit team
	Grievance co-ordinator	1 member of the finance team
		2 members of the policy team
		Grievance redressal manager
		1 Project manager
		1 member of the data analytics team
**Insurance Company**	Project manager	
**Third-party agencies/ implementation support agencies**	3 Project heads	4 State coordinators
	2 Claim processing doctors	2 Claim processing doctors
	1 Beneficiary identification system executive	1 Beneficiary identification system executive
	Zonal coordinator	Project manager
	District TPA manager	
**District Implementation Unit**	2 Civil Surgeons	3 Chief Medical Officers
		2 Additional Chief Medical Officer
		5 members of DIU team
**Empanelled hospitals**	9 Ayushman mitras	7 Ayushman mitras
	7 Administrators	9 Administrators
	2 Health care providers	3 Health care providers

TPA- third-party agency, DIU- district implementation unit

Supplementary Table 3. Break-up of annual estimated administrative costs for Uttar Pradesh in the first year of the scheme from September 2018

Cost head	State level	District level	Total
Human resources recruited for PMJAY[a]	30,460,000 INR (421,300 USD)	144,000,000 INR (1,991,701 USD)	174,460,000 INR (2,413,001 USD)
Contracting of implementation support agencies [b]	41,544,095 INR (574,607 USD)	-	41,544,095 INR (574,607 USD)
Transportation costs[c]	6,000,000 INR (82,988 USD)	27,000,000 INR (373,444 USD)	33,000,000 INR (456,432) USD)
Materials costs[c]	11,000,000 INR (152,144 USD)	16,200,000 INR (224,066 USD)	27,200,000 INR (376,210 USD)
Miscellaneous[c]	60,000,000 INR (829,876 USD)	10,800,000 INR (149,378 USD)	70,800,000 INR (979,253 USD)
**Total**	**149,004,095 INR** (2,060,914 USD)	**198,000,000 INR** (2,738,589 USD)	**347,004,095 INR** (4,799,503)

[a] Based on the sanctioned positions and planned remuneration.
[b] Based on actual contract value, cumulative figure provided for state and districts
[c] Estimates provided by state government officials
Source – Data obtained from the State Health Agency of Uttar Pradesh
